# Magneto-optical micromechanical systems for magnetic field mapping

**DOI:** 10.1038/srep31634

**Published:** 2016-08-17

**Authors:** Alain Truong, Guillermo Ortiz, Mélissa Morcrette, Thomas Dietsch, Philippe Sabon, Isabelle Joumard, Alain Marty, Hélène Joisten, Bernard Dieny

**Affiliations:** 1 Univ. Grenoble Alpes, INAC-SX, F-38000, Grenoble, France; 2 CEA, INAC-SX, F-38000, Grenoble, France; 3 CNRS, SX, F-38000, Grenoble, France; 4 CEA, LETI, Minatec Campus, F-38000, Grenoble, France

## Abstract

A new method for magnetic field mapping based on the optical response of organized dense arrays of flexible magnetic cantilevers is explored. When subjected to the stray field of a magnetized material, the mobile parts of the cantilevers deviate from their initial positions, which locally changes the light reflectivity on the magneto-optical surface, thus allowing to visualize the field lines. While the final goal is to be able to map and quantify non-uniform fields, calibrating and testing the device can be done with uniform fields. Under a uniform field, the device can be assimilated to a magnetic-field-sensitive diffraction grating, and therefore, can be analyzed by coherent light diffraction. A theoretical model for the diffraction patterns, which accounts for both magnetic and mechanical interactions within each cantilever, is proposed and confronted to the experimental data.

Magnetic field mapping techniques have continuously been developed due to the necessity for determining the spatial components of local magnetic fields in many industrial applications and fundamental research^1^,^2^,^3^,^4^. A well-established tool for obtaining the spatial distribution of magnetic fields is the magneto-optical imaging (MOI) method, which makes use of so-called MOI films such as ferrite garnet films with uniaxial or planar anisotropy^5^,^6^, and nanoparticle Bitter films^7^,^8^,^9^,^10^,^11^. A MOI film placed on a magnetized surface will have its magnetic domain structure reorganized in accordance with the local intensity of the magnetization or stray field produced by the analyzed material. However, the method is non-quantitative and depositing a MOI film is an invasive process that leaves traces after removal. Other field mapping techniques are based on scanning, such as superconducting quantum interference device (SQUID)^12^,^13^, Hall bar^14^,^15^,^16^, magnetic force^17^,^18^, and scanning magnetoresistive microscopy^19^. Despite being highly sensitive, scanning techniques are slow and each of them has its own drawback. For instance, SQUID microscopy operates at low temperatures, and magnetoresistive sensors operate with dc currents, thus possibly introducing heat and magnetic fields that can alter the sample. Due to their long spin coherence time, negatively charged nitrogen-vacancy centers in diamond are also promising for magnetometry^20^,^21^,^22^,^23^. However, nanometer scale proximity to the surface of the diamond film is necessary and is limited by the charge stability of the surface state acceptors. Several factors are considered for sensors^12^ such as spatial resolution, sensitivity, linear response, required proximity to the sample, as well as the ability to filter noise and to measure without bringing in any perturbations to the sample. The existing techniques usually combine several of the desired features and in many cases they are found to be complementary. In this paper, we report magnetic field mapping by use of a structure based on organized arrays of close-packed mobile magnetic elements. Each element is a magnetic cantilever with microscopic length and nanometer scale thickness. When placed in proximity to magnetized materials, the cantilevers are deflected depending on the intensity and direction of the stray fields. Due to a local change of reflectivity induced by the stray field, the field lines generate a contrast, which is even visible to the naked eye on the surface of the mapping device. Such magneto-optical surface offers the advantage of being a passive and easy-to-use device, since neither power source nor sample preparation is required. It is also directly sensitive to magnetic torques rather than magnetic field gradients. The goal of such device is to map non-uniform fields by extracting from each cantilever the local intensity and direction of the field. Uniform magnetic fields can however be used to calibrate and quantify the sensitivity of the mapping device. Since all cantilevers deflect the same way in a uniform magnetic field, the device is optically equivalent to a magnetic-field-sensitive diffraction grating. The field mapping technique presented here opens up possibilities for applications such as microcracks detection by measuring current distributions in metals or detection of ferromagnetic pigments used in security applications.

## Results

### Visualizing field lines with organized arrays of magnetic cantilevers

**Figure 1:**
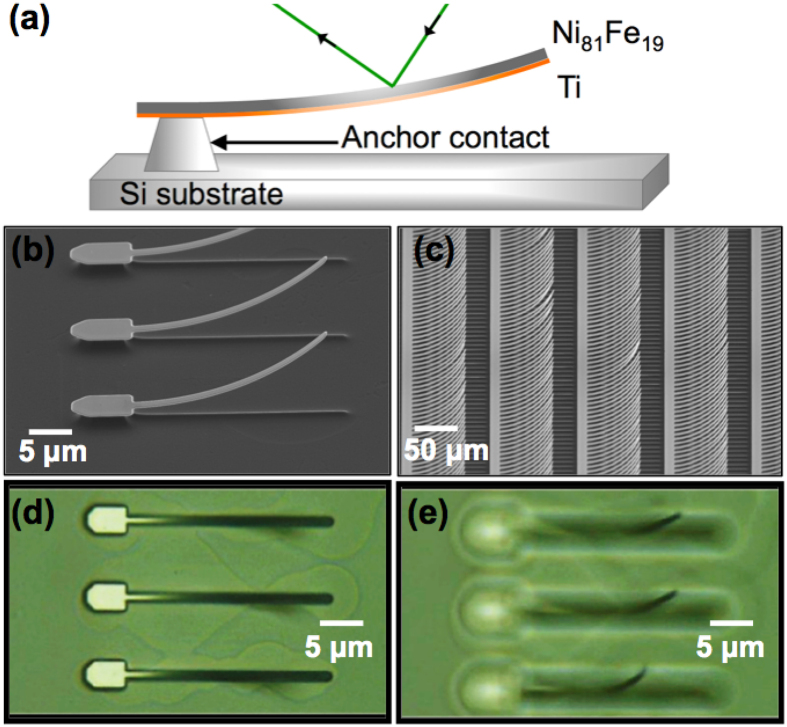
(**a**) Schematic of one magnetic microcantilever. (**b,c**) SEM images of arrays of cantilevers of several size and density. The influence of a magnetic field causes a curvature. Optical microscope images are taken (**d**) without an external magnetic field and (**e**) with a field. The magnetic field is applied by placing a NdFeB permanent magnet under the substrate of the sample.

The magneto-optical surfaces are developed by a top-down technique on silicon (Si) substrates. A Ma-N 2403 negative tone photoresist layer is deposited by spin-coating and patterned after the rectangular-section beam shape of the microelements by UV lithography. Then, a 100 nm layer of permalloy (Ni_81_Fe_19_) is deposited by sputtering on a 3 nm titanium (Ti) layer. The purpose of the Ti layer is to promote the adhesion of the permalloy layer on Si. Different lengths and spacings between each beam can be achieved with different types of masks. The remaining photoresist is then lifted-off and the Si substrate is isotropically etched by reactive ion etching using a sulfur hexafluoride (SF_6_) plasma, which liberates the beams. The beams have one end, which is patterned with wider dimensions to ensure that the SF_6_ plasma does not entirely etch the Si underneath, so that the anchor contact (Fig. 1a) can be formed. Once the substrate is removed, the free-standing part of the cantilevers bends because of the residual stress^24^,^25^ due to the elevated deposition temperature (~100 °C) and the difference of thermal expansion coefficients between Si (2.56 × 10^−6^ K^−1^), Ti (8.6 × 10^−6^ K^−1^) and permalloy (12 × 10^−6^ K^−1^). Once liberated, the magnetic microelements are free to be actuated by an external magnetic field. Typical scanning electron microscope (SEM) images of arrays of magnetic cantilevers are shown in Fig. 1b,c. Optical microscopy coupled with a high-speed camera system allows to observe the real-time spontaneous motion of the beams when they are exposed to a magnetic field (Fig. 1d,e). In our case, the permalloy layers are vacuum-deposited. So, to ensure the stability of the magnetic cantilevers against oxidation when exposed to the air, a 1 or 2 nm layer of a metal that gets passivated in the air (Al, Ta) can be grown before taking the permalloy film out of the vacuum chamber. Such thin film does not significantly alter the Young’s modulus of the cantilever.

**Figure 2:**
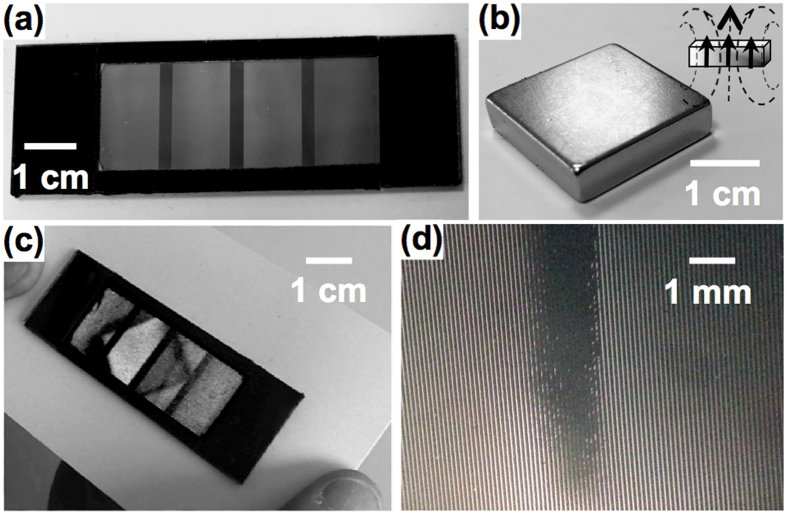
(**a**) Test device made of four different arrays of cantilevers of different size (2 *μ*m wide and 30, 60, 40, and 80 *μ*m long respectively, from left to right) on a Si wafer, similar to the one magnified in Fig. 1d,e. (**b**) The NdFeB permanent magnet is (**c**) placed under the test device, which is perfectly opaque. The field lines form contrasted regions, which remind of shape of the magnet. (**d**) Close-up view by optical microscopy of a contrasted area.

To test this concept of field mapping, an experiment is first conducted at macroscopic scale. A test device (Fig. 2a) is placed over a NdFeB permanent magnet (Fig. 2b). A striking result is that the field lines produce contrasted regions that are visible to the naked eyes on the magneto-optical surface (Fig. 2c,d). The surface of the device recovers its initial state after the field is removed, so the effect is reversible. The white light reflects differently depending on the local deflection of each cantilever caused by the stray field, thus giving rise to the dark regions that recreate the shape of the magnet. In a second step, in order to quantify the strength of the observed stray field, we employ optical diffraction by coherent light in a uniform magnetic field.

### Theoretical model using Hamiltonian mechanics

**Figure 3:**
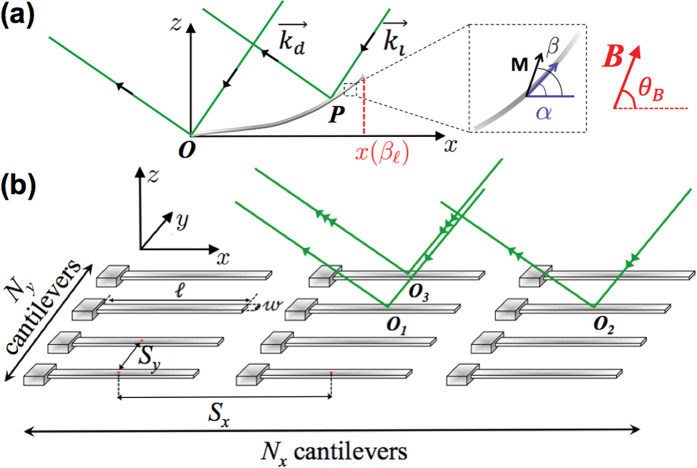
Coordinate system used for the calculations. (**a**) The beam and the magnetization have a local angle *α* and *β* with the *x*–axis, respectively. The external magnetic field 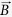 is applied with an angle *θ*_*B*_ with respect to the *x*–axis. The optical phase differences are calculated from the clamped origin point *O*. (**b**) The dimensions of a beam are noted 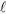, *h, w* for the length, the thickness and the width, respectively. The geometric centers of the cantilevers are separated by the distances *S*_*x*_ and *S*_*y*_ in the *x* and *y* directions, respectively.

Considering the arrays of microcantilevers as an actuable diffraction grating, we establish a theoretical model that relates the diffraction pattern to the deflection caused by a magnetic field with a certain magnitude and direction. Expressing the shape of the deflected cantilevers by an adequate function is required to obtain the diffraction patterns analytically. The coordinate system and the notations related to the array of cantilever are defined in Fig. 3. For the theoretical model, the deformation occurs in the (*zOx*) plane, and the cantilevers are considered to be long and slender beams that are made of isotropic materials. Those conditions being met, the model accounting for the internal stress caused by the action of an external magnetic field on the magnetic cantilever can be developed in the frame of the Euler-Bernoulli beam theory^26^. Here, we choose an energy approach to describe the interplay between the magnetic and the mechanical properties of the materials. The total energy per unit length of the beam, expressed as a function of the curvilinear coordinate *s*, includes the bending energy *E*_*c*_(*s*), and the magnetic energy *E*_*m*_(*s*), which contains the Zeeman term due to the external field and the shape anisotropy term. *α* and *β* are the local angles between the neutral axis of the beam and the *x*–axis, and between the magnetization and the *x*–axis, respectively. Since the beams are made of permalloy, the magnetocrystalline anisotropy and the effects of magnetostriction are negligible. Therefore, the uniaxial shape anisotropy along the neutral axis of the beam is the dominant form of magnetic anisotropy. The influence of the exchange and dipolar interactions on the deformation are neglected. For a given external load on the cantilevers, the functional minimization of the total energy with respect to the function *α*(*s*) leads to the relation between d*s* and d*α*.


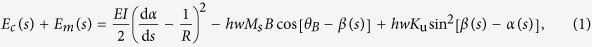



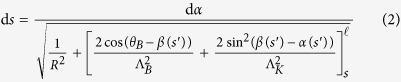


where 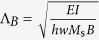 and 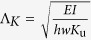 are characteristic lengths that are related to the external magnetic field and the uniaxial anisotropy, respectively. *M*_s_ is the saturation magnetization of permalloy, *K*_u_ is the uniaxial anisotropy energy coefficient, *θ*_*B*_ is the magnetic field angle, *E* is the combination of the Young’s moduli of both permalloy and Ti layers according to the Reuss rule of mixture^27^, 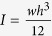 is the centroidal bending stiffness. In order to account for the initial bending of the cantilevers, a homogeneous residual stress is included in the bending energy term by giving a constant initial radius of curvature *R* to the beam. From equation (2), one can express the shape of the beam with a parametric curve (see the Methods section). For the calculations, *R* and *θ*_*B*_ are fitting parameters.

The values of *α* and *β* at the free end of the cantilever are noted as 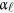 and 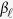, respectively. A consequence of this model is that *α* and *β* are single-valued functions of *s*. Thus, for a given length 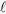 of the beam, applying an external applied magnetic field with a certain magnitude *B* and a certain angle *θ*_*B*_ will result in a unique set of values for the pair 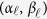.

Equation (1) implies that the external field applies a torque on the magnetization of the material, which in turn exerts a torque on the easy axis of the ferromagnet, thus causing the beam to bend. Although the uniaxial shape anisotropy would normally favor an alignment of the magnetization along the neutral axis of the cantilever, the combined presence of both bending and Zeeman energies can, in principle, allow the magnetization to be oriented in directions non-parallel to the tangent to the beam. To verify this hypothesis, the equilibrium angle of the magnetization with respect to the neutral axis of the beam is determined at each point on the cantilever by minimizing the magnetic energy with respect to *β*, i.e. ∂*E*_*m*_/∂*β* = 0, which results in the following relation:


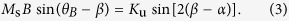


**Figure 4:**
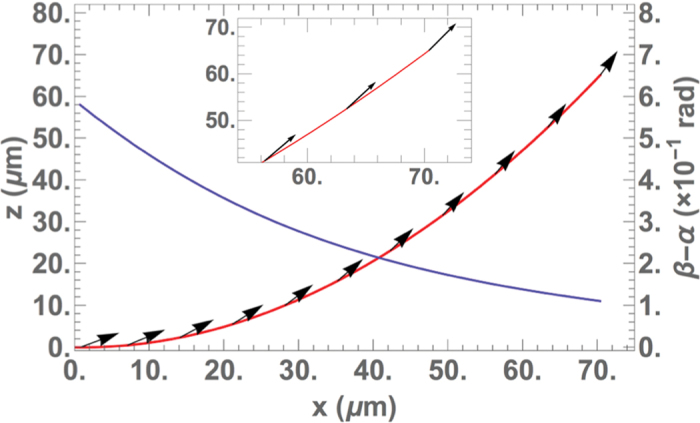
The beam is deflected under an external field of 700 Oe (red curve). The calculated magnetization vectors are represented by black arrows. The angle (*β* − *α*) between the magnetization and the neutral axis decreases along the beam (blue curve).

Equation (3) can also be seen as an equilibrium between the torque exerted by the external field on the magnetization and that exerted by the magnetization on the easy magnetization axis. The calculations are done with *M*_s_ = 800 emu.cm^−3^, *K*_u_ = 43000 erg.cm^−3^, values for permalloy thin films^28^,^29^,^30^ at 300 K, and the beams have a length 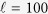 *μ*m and width *w* = 2 *μ*m. Figure 4 represents the shape of a 100 *μ*m long beam deflected under a magnetic field of 700 Oe, with a field angle *θ*_*B*_ = 1.36 rad, and the stable equilibrium orientation of the magnetization calculated at several positions on the beam. It is clear from Fig. 4 that the magnetization vectors slightly point out of the beam. The effect becomes weaker as one approaches the free end, which is consistent with a decreasing bending torque as *x* increases, since *β* gets closer to *θ*_*B*_.

## Discussion

The magneto-mechanical model computes the cantilever shape that best fits the intensity profile of the coherent light diffraction patterns measured experimentally in the Fraunhofer conditions. The theoretical wave diffraction patterns are obtained by calculating the total diffracted intensity for a given curvature. The total intensity of the diffracted light, at coordinates (*x*_*M*_, *y*_*M*_) on the observation plane, is the product of the interference grating term 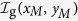 by the Fraunhofer diffraction term 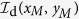, which is adjusted to account for the curvature. Using the notations in Fig. 3, the phase difference between the waves diffracted at *O*(0, 0, 0) and *P*(*x, y, z*(*x*)) is given by 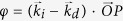, for an incidence quasi-normal to the magneto-optical surface:


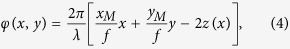


where *f* is the focal length of the converging lens that forms the diffraction pattern in the observation plane, 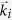 and 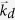 are the incident and diffracted wave vectors, respectively, and *λ* = 533 nm is the wavelength of the laser.


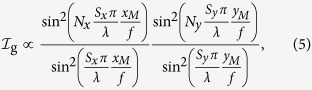



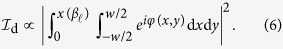


**Figure 5:**
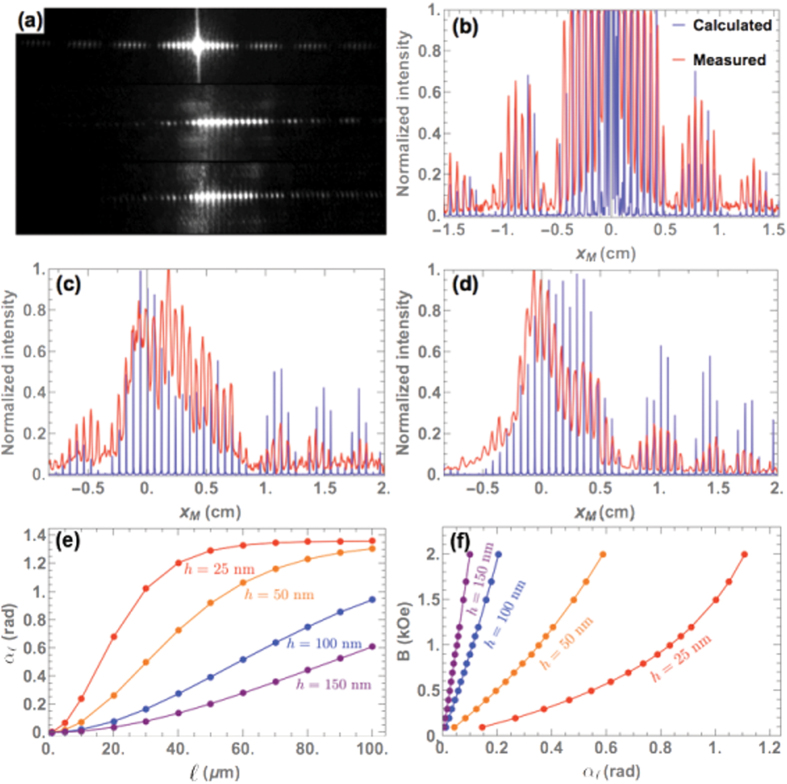
(**a**) The diffraction patterns measured for three different configurations in the *x*_*M*_ direction at a fixed value of *y*_*M*_: array of flat cantilevers (top), array of bent cantilevers under a magnetic field of 700 Oe (center), and 800 Oe (bottom). The corresponding intensity profiles are shown: (**b**) flat configuration, (**c**) under a field of 700 Oe, and (**d**) 800 Oe. By inputting different values of thickness *h* in the model, we obtain: (**e**) the predicted values of 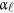 as a function of the total length 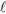 of the beam at *B* = 700 Oe, and (**f**) the relation between the values of magnetic field and the deflection for 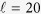 *μ*m.

The spacing of the grating and the number of cantilevers are *S*_*x*_ = 112 *μ*m, *N*_*x*_ = 20 and *S*_*y*_ = 4 *μ*m, *N*_*y*_ = 100 in the *x* and *y* directions, respectively. When the cantilevers are flat, the array gives an optical response corresponding to a symmetric pattern (Fig. 5a,b). When the structure is bent, the diffraction pattern becomes asymmetric because the envelope of the optical signal, determined by 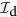, changes when the magnitude of the external field varies (Fig. 5a,c,d). The results in Fig. 4 are obtained with the fitting parameters *R* = 155 *μ*m and *θ*_*B*_ = 1.36 rad. Then, by incorporating the mathematical expression of the shape of the cantilevers in equation (6), we found a relatively good match between the measured optical signals and the calculated diffracted intensities. The data fittings in Fig. 5c,d yield the magnitude of the magnetic field *B*: 700 Oe and 800 Oe, respectively. A gaussmeter is placed under the sample to confirm the value of the field. Consequently, the above-mentioned theoretical approach appropriately models the shape of the beams bent under the influence of a certain stray field. In addition, this model can also be used to predict the most optimal cantilever dimensions to improve the sensitivity and the resolution for magnetic field mapping. The quantity 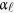 is the highest value of *α*(*s*) for a given length 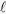, hence it measures how much a cantilever can be deflected by a field. Shorter beams make it possible to build denser arrays for better spatial resolution but reduce the ability of the beam to deflect. However, thinner beams can be deflected more easily, therefore at an equal amplitude of external field, they have a higher sensitivity than thicker beams (Fig. 5e,f). A possible way to enhance the sensitivity of the device is to fabricate cantilevers with non-uniform thicknesses: smaller thicknesses around the clamped end will reduce the overall stiffness, while higher thicknesses near the free end will increase the magnetic torque. The fabrication process would nonetheless be more complexe. Tuning the centroidal stiffness *I* by changing the geometry of the cantilevers in the *y* direction can be a more feasible alternative.

In summary, we have shown that a magneto-optical surface made up of organized arrays of magnetic-field-actuable cantilever beams can react to the direction and the magnitude of a stray field emanated from a magnetized material. At the macroscopic scale, the effects can be visible to the naked eye, but they can also be visualized at the microscopic scale by optical microscopy, which allows a real-time two-dimensional imaging of the magnetic field distribution. The arrays of magnetic cantilevers are directly sensitive to magnetic torques, rather than magnetic field gradients. This property is attractive because measuring the deflection of the cantilevers is equivalent to measuring the magnetic torque applied on it, hence magnetic fields can be quantified. An interesting challenge is to further improve the spatial resolution and the sensitivity by changing the dimensions or the geometry of the beams. Beam thicknesses of a few tens of nanometers offer higher sensitivity, while lengths as short as a few microns stiffen the beams, which goes against optimizing the sensitivity. Augmenting the sensitivity of the device by changing the geometry of the cantilevers and in particular by using beams of non-uniform thickness is a path to explore. The upper and lower limit depends on the mechanical properties of the cantilevers. A quantitative description of the relationship between applied field and cantilever deflection is given in relation to Fig. 5e,f. With the geometry described in this paper, fields in the range 0.1 to 2 kOe can be detected and mapped. In principle, the sensitivity of this approach could be much increased by using vertically suspended magnetic cantilevers, which would hang below the substrate. In this case, the cantilever could be made much thinner since they would not have to support their own weight. As a result, they would react to much lower field than those described in Fig. 3. In this case, magnetic fields of the order of the earth field could be detected. For the type of foreseen applications, there is no interest in trying to further enhance the sensitivity of the cantilevers since they are expected to be used in presence of the earth field. The drawback of this technique is however its sensitivity to vibrations and the volume required not for the device itself but for the optical readout setup.

## Methods

### Distributed magnetic torque on the magnetic beams

The magneto-mechanical model is developed in the frame of Hamiltonian mechanics. The total energy per unit length of the beam corresponds to a Lagrangian 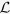 given in terms of generalized coordinate *α* and generalized velocity 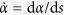. For a given external load imposed by the applied magnetic field, the Legendre transform of 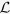 is invariant along the beam:


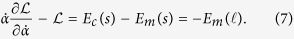


The equation above leads to the following first order differential equation:


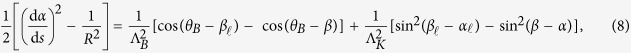


Solving the equation analytically is not trivial because the variable *s* is contained in both functions *α* and *β*. Since 
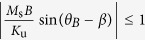
, equation (3) allows to isolate the variables *α* and *β*, which eliminates *α* in the expression of d*s* in equation (2): d*s* = Λ_*K*_*g*(*β*)d*β*, with


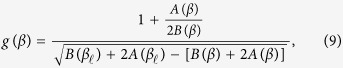


where 
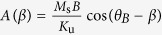
 and 
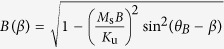
. The shape of the cantilever is given by a parametric curve by calculating 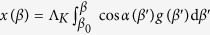 and 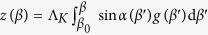, where *β*_0_ = *β*(*s* = 0) is found with equation (3) using the boundary condition at the clamped end *α*(*s* = 0) = 0. Since *s* is a single-valued function of *β, β*(*s*) is found by inverting *s*(*β*). By inserting the values of *β*(*s*) in equation (2), *α*(*s*) is determined, thus allowing to calculate the difference of angles *β*(*s*) − *α*(*s*) along the beam.

## Additional Information

**How to cite this article**: Truong, A. *et al*. Magneto-optical micromechanical systems for magnetic field mapping. *Sci. Rep.* **6**, 31634; doi: 10.1038/srep31634 (2016).
